# A case report of an Egyptian family with familial hypercholesterolemia and an exonic LINE‐1 insertion in *LDLR*

**DOI:** 10.1002/mgg3.2410

**Published:** 2024-03-04

**Authors:** Yongjun Song, Reham Abdel Haleem Abo Elwafa, Omneya Magdy Omar, Go Hun Seo, Hane Lee

**Affiliations:** ^1^ 3billion, Inc. Seoul South Korea; ^2^ Department of Clinical Pathology, Faculty of Medicine Alexandria University Alexandria Egypt; ^3^ Department of Pediatrics, Faculty of Medicine Alexandria University Alexandria Egypt

**Keywords:** exome sequencing, familial hypercholesterolemia, LDLR, mobile element insertions, rare mendelian disorders

## Abstract

**Background:**

Familial hypercholesterolemia (MIM: PS143890) is a genetic disorder characterized by an increase in blood cholesterol. *LDLR* is one of the genes which their defect contributes to the disorder. Affected individuals may carry a heterozygous variant or homozygous/compound heterozygous variants and those with biallelic pathogenic variants present more severe symptoms.

**Method:**

We report an Egyptian family with familial hypercholesterolemia. Both the proband and parents have the disorder while a sibling is unaffected. Exome sequencing was performed to identify the causal variant.

**Results:**

LINE‐1 insertion in exon 7 of *LDLR* was identified. Both parents have a heterozygous variant while the proband has a homozygous variant. The unaffected sibling did not carry the variant.

**Discussion:**

This insertion may contribute to the high prevalence of hypercholesterolemia in Egypt and the finding underscores the importance of implementing mobile element insertion caller in routine bioinformatics pipeline.

## Graphical abstract

Familial hypercholesterolemia (MIM: PS143890) is a genetic disorder characterized by an increase of blood cholesterol. The LINE‐1 mobile element insertion was identified in an Egyptian family with familial hypercholesterolemia. This insertion may contribute to the high prevalence of hypercholesterolemia in Egypt and the finding underscores the importance of implementing mobile element insertion caller in routine bioinformatics pipeline.

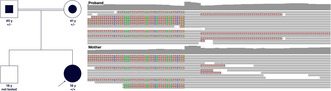

## 1 INTRODUCTION

Familial hypercholesterolemia (FH, MIM: PS143890) is a genetic disorder characterized by an increase of total cholesterol (TC) and low‐density lipoproteins‐cholesterol (LDL‐C). It can be caused by defects in genes involved in cholesterol regulations such as the low‐density lipoprotein receptor gene, *LDLR* (MIM: 606945). LDLR, associated with familial hypercholesterolemia type I (MIM: 143890) (Hobbs et al., 1992), mediates endocytosis of cholesterol‐rich low‐density lipoprotein. Disruption of *LDLR* reduces the number of functional LDLRs on the cell surface and decreases LDL cholesterol endocytosis, resulting in an increase of serum LDL‐cholesterol and total cholesterol level. Increased serum cholesterol level causes tendon xanthoma, xanthelasma, corneal arcus, and accelerates atherosclerosis. Affected individuals may carry a heterozygous pathogenic variant or homozygous/compound heterozygous pathogenic variants in *LDLR* and those with biallelic pathogenic variants have much higher levels of LDL‐C than those with monoallelic pathogenic variant and hence have higher risks of clinical manifestations of the disease such as having a coronary artery disease at a younger age.

Numerous loss‐of‐function (LoF) variants in *LDLR* including premature termination variants, missense variants, and structural rearrangements such as large deletions/duplications and mobile element insertions are reported to cause FH (Fokkema et al., 2011; Leigh et al., 2017). Mobile elements (ME), or transposable elements, are DNA fragments that “move” around the genome through transcription and insertion. These mobile element insertions (MEI) can insert within or near a gene and disrupt gene function or alter gene expression to cause a disease. Three types of MEs are known to remain active: long interspersed nuclear element 1(LINE‐1), *Alu* sequence and SVA elements. Together, these mobile elements represent more than 28% of the human genome (Payer & Burns, 2019). It is challenging to find MEIs as disease‐causing variants using conventional sanger sequencing or even next‐generation sequencing (NGS) but not impossible. As NGS variant calling algorithms improve and long‐read sequencing becomes available, more MEIs are being discovered as causal variants (Gardner et al., 2017; Watson et al., 2021). Here, we present a case of an Egyptian family with FH caused by a unique LINE‐1 insertion in a coding exon of the *LDLR* gene.

## 2 MATERIALS AND METHODS

### 2.1 Exome sequencing and variant interpretation

Genomic DNA was extracted from whole blood specimens using QIAamp DNA Blood Mini Kit (250) (Qiagen #51106) following the manufacturer's instructions. Exome capture was performed using IDT xGen Exome Research Panel v2.0 (Integrated DNA Technologies, Coralville, Iowa, USA) and sequencing was run on NovaSeq 6000 system (Illumina, San Diego, CA, USA). The base call (BCL) sequence files were converted and demultiplexed to FASTQ files using bcl2fastq v2.20.0.422 (RRID:SCR_015058). Sequence reads were aligned to the human reference genome (GRCh37/hg19 from NCBI, February 2009) and revised Cambridge Reference Sequence (rCRS) of the mitochondrial genome using BWA‐mem 0.7.17 to generate BAM files (Li, 2013). BAM files were processed following the GATK best practices (GATK v.3.8) (Van der Uwera & O'Connor, 2020) for single‐nucleotide variants (SNV) and small insertions/deletions (INDEL) variant calling to generate VCF files (DePristo et al., 2011; McKenna et al., 2010). CoNIFER (Krumm et al., 2012) and 3bCNV, an in‐house‐developed tool that identifies exons with significant differences in depth‐of‐coverage (DOC) of a sample compared to a control group (manuscript in preparation), were used for copy number variant (CNV) calling. AutoMap v1.2 (Quinodoz et al., 2021) was used for region of homozygosity (ROH) detection from the VCF file. The variant analysis was performed using an internally developed system, EVIDENCE (Seo et al., 2020), to annotate and classify variants based on the American College of Medical Genetics and Genomics (ACMG) and the Association for Molecular Pathology (AMP) guidelines (Richards et al., 2015). The variants were filtered and prioritized before being manually curated by medical geneticists. All clinically significant variants were manually examined using Integrative Genomics Viewer (IGV, Version 2.9.4) (Thorvaldsdóttir et al., 2013) to check the quality of the variant and for CNVs, to further delineate the nature of the call. For the calls that were deemed technically true, an internal variant database of 24,747 exome sequencing data that was analyzed using the same bioinformatics pipeline was searched to see how common the variant is.

## 3 RESULTS

### 3.1 Patient

**FIGURE 1:**
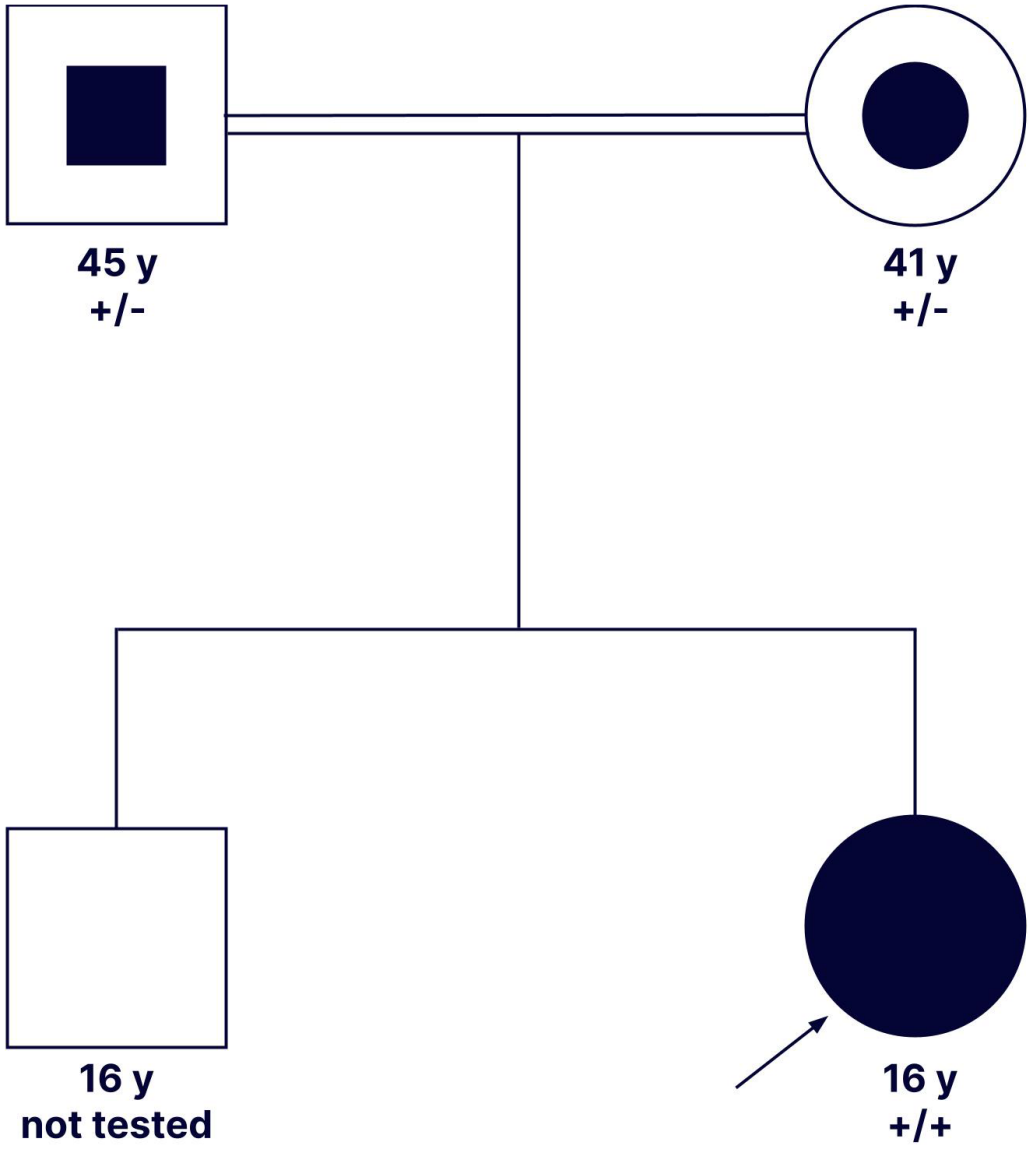
Family pedigree. Arrow: proband. Circles: female, squares: male. Filled circles/squares: severely affected individuals. Partially filled circles/squares: mildly affected individuals. Empty circles/squares: unaffected individuals. Double line: consanguineous mating. +/−: heterozygous for the variant. +/+: homozygous for the variant.

The proband, a 14‐year‐old female, presented xanthomatosis on her forearms and buttocks, as well as hypercholesterolemia (LDL‐C: 625.6 mg/dL, normal: 50–100 mg/dL). She started to present these symptoms at the age of four. While both of her parents, who are second cousins, had mild hypercholesterolemia (Father LDL‐C: 268.2 mg/dL, Mother LDL‐C: 269 mg/dL), her brother did not present any symptoms (LDL‐C: 101.6 mg/dL, Figure 1 and Table 1). Lipid profiles were conducted for all four family members, revealing that the proband had severe hypercholesterolemia (LDL‐C levels >300 mg/dL) with a total lipid of 1224 mg/dL, TC of 689 mg/dL, TG of 127 mg/dL, HDL‐C of 38 mg/dL, non‐HDL‐C of 651 mg/dL, LDL‐C of 625.6 mg/dL, TC/HDL ratio of 18.13, and LDL/HDL ratio of 16.46 (Table 1). She was prescribed simvastatin (40 mg) and ezetimibe (10 mg), which helped to manage her hypercholesterolemia. No abnormal findings were detected during the fundus examination.

**TABLE 1:** Lipid profile of the family.

Family members	TL (mg/dL)	TC (mg/dL)	TG (mg/dL)	HDL‐C (mg/dL)	Non‐HDL‐C (mg/dL)	LDL‐C (mg/dL)	TC/HDL ratio	LDL/HDL ratio
Proband	1224	689	127	38	651	625.6	18.13	16.46
Father	613	325	84	40	285	268.2	8.13	6.71
Mother	657	343	95	55	288	269	6.24	4.89
Brother	420	173	107	50	123	101.6	3.46	2.03

*Note*: Reference ranges: TL: up to 800 mg/dL; TC: up to 200 mg/dL; TG: up to 200 mg/dL; HDL‐C: favorable >55, moderate risk 35–55, high risk <35 mg/dL; non HDL‐C: optimal <130, near optimal 130–160, borderline high 160–190, high risk 190–220, very high risk >220 mg/dL; LDL‐C: favorable <130, moderate risk 130–159, high risk > = 160 mg/dL; TC/HDL ratio: favorable 3.3–4.4, average 4.4–7.1, moderate 7.1–11.0, high risk >11.0; LDL/HDL ratio: favorable 0.5–3.0, moderate 3.0–6.0, high risk >6.0.

The patient and her parents were consented for a genetic study approved by the Alexandria Faculty of Medicine Ethics Committee (IRB code 00012098‐FWA: No. 00018699. Ethics approval number: No. 0305926).

### 3.2 Exome sequencing and data analysis

The proband exome sequencing data showed 98.8% of the targeted regions having at least 20X DOC. The proband had 3.43% ROH across the genome, being consistent with her parents being 2nd cousins in a family with multiple consanguineous marriages. No clinically significant SNV/INDEL were identified that can explain the patient's phenotype. All the coding regions of the eight genes associated with FH (MIM: PS143890), *LDLRAP1, PCSK9, APOA2, APOB, GHR, GSBS, EPHX2, LDLR*, were covered well without any deletions or duplications except for the *LDLR* gene that had an unusual decrease of coverage (*z*‐score = −5.19) in exon 7 (NM_000527.5) with a small hump in the middle of the exon.

### 3.3 Identification of the LINE‐1 insertion

**FIGURE 2:**
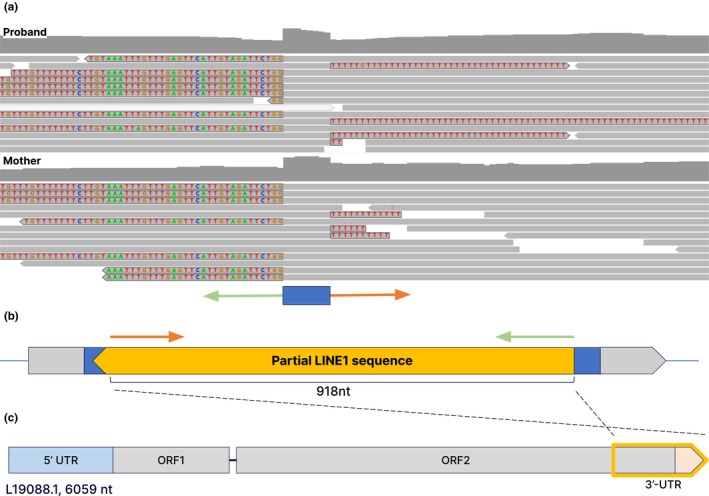
LINE‐1 insertion of the family presented. (a) IGV snapshot displaying soft‐clipped sequences. The soft‐clipped reads on the left‐end are mapped to the 5141st nucleotide of the LINE‐1 sequence L19088.1. When considered with the poly‐T sequences on the right‐end, this soft‐clipped reads on the left‐end indicate insertion of a truncated and reversed L1 sequence with a poly A tail. Unlike the snapshot of the proband, half of the reads of the mother is not soft clipped in either end indicating heterozygous state of the MEI. The paternal result displayed the same pattern. (b) An overview of the 918‐nt long insertion is shown, including sequences of interest colored as follows: gray represents exon 7 of *LDLR*, blue represents small duplicated sequences (ATGACCTT), and yellow region is the inserted reversed partial LINE‐1 sequence. (c) Reference LINE‐1 sequence L19066.1. The inserted sequence is highlighted in a yellow box.

The coverage drop in exon 7 of the *LDLR* gene was further investigated. All sequence reads spanning the small hump in the middle of the exon had soft‐clipped sequences at either end. The soft‐clipped sequence on the right‐end of the reads were poly‐Ts and the sequences on the left‐end of the reads were “ATGGGGTTGTTTGTTTTTTTCTTGTAAATTTGTTTGAGTTCATTGTAGATTCTGG” (Figure 2). When the latter sequence was queried to the human reference genome, it was found to blat to hundreds of places, suggesting that this was a repeat sequence. All these regions were annotated as LINE‐1 sequences in RepeatMasker (Smit et al., 2013–2015). To find exactly which part of the LINE‐1 sequence the soft‐clipped sequence was from, we aligned it to the LINE‐1 reference sequence (GenBank accession number: L19088.1) to find that it was from base 5141 to 5211. Predicted size of the partial LINE‐1 sequence inserted was 918 bp: from base 5141 to the last base 6059 of LINE‐1. Because all reads had soft‐clips at either end, we could assume that the insertion was homozygous (Figure 2) and the sequence of the unusual hump was “ATGACCTT,” representing the “target site duplication,” a hallmark of MEI. The same pattern of soft clipped reads was observed in the parental data, except that in the parents, only about half the reads had soft clips, suggesting that they were heterozygous for the insertion. This insertion was not seen in any of the 24,747 internal exome sequencing data, including the 2174 patients' data from Egypt.

## 4 DISCUSSION

FH is diagnosed based on the lipid levels accompanied by family history, physical examination, and genetic analysis. While there are multiple well‐defined clinical diagnostic tools such as “US Make Early Diagnoses Prevent Early Deaths Program Diagnostic Criteria” to diagnose FH, some patients still may not be adequately diagnosed just based on the clinical criteria. Also, even if a clinical diagnosis is made with confidence, identifying the underlying genetic cause could help the patient and the family members to receive more precise clinical management. Therefore, many countries are incorporating genetic testing as a part of the screening strategies (Bouhairie & Goldberg, 2015) and expanding the spectrum of genetic variants is crucial in designing a better screening program.

Here, we present a case with a novel homozygous LINE‐1 insertion within a coding exon of the *LDLR* gene. Numerous disease‐causing *LDLR* variants are reported: 2295 pathogenic/likely pathogenic variants were reported in ClinVar as of November 2023, which includes two Alu element insertions (ClinVar: VCV001074682, VCV001069978). However, LINE‐1 insertion in *LDLR* has never been reported. Although it could not be validated at the transcript or protein level, this insertion is expected to disrupt the function of the gene (Hancks & Kazazian, 2012) which is consistent with the known disease mechanism. The variant was inherited from the parents who are each heterozygous for the variant. As expected, the heterozygous parents had a relatively milder phenotype compared to the homozygous proband who presented with doubled TC and LDL‐C level compared to the parents, supporting pathogenicity of the insertion.

In this case, the variant was found in one of the regions of homozygosity and we could hypothesize that the insertion was inherited from a common ancestor of the family and FH runs in the family. Interestingly, the prevalence of FH in Egyptian patients with atherosclerotic cardiovascular disease (ASCVD) is estimated to be 17% (Reda et al., 2021) while overall prevalence of FH in ASCVD patients is predicted to be 5.88% worldwide (Hu et al., 2020). However, there is no report of pathogenic *LDLR* variants that are common in the Egyptian population. To see if this MEI could be one of the common variants in the Egyptian population with FH, we searched through our internal database of 24,747 exome data including 2174 patients from Egypt but did not find any samples carrying the same MEI variant.

Approximately, 30% of the genome is composed of repeat sequences such as SINE and LINE and it imposes difficulties to map short‐read sequences containing a fragment of MEs, either reference or non‐reference. In many laboratories, MEI discovery is not part of the main bioinformatics pipeline yet (Torene et al., 2020) because it needs a separate caller, specifically designed to identify these variants. Here, we were able to find the MEI because the disrupted exon had significantly lower DOC. This is one way to discover MEIs as there is a specific pattern of mapped sequence reads when there is an MEI. Also, there are tools designed to call non‐reference MEIs such as MELT (Gardner et al., 2017), SCRAMble (Torene et al., 2020), that take discordant reads and align to known ME sequences to discover short sequence reads containing non‐reference MEIs. A couple of studies that performed large‐scale MEI calling on exome sequencing data report diagnostic rates of 4/9738 (0.04%) (Gardner et al., 2017), and 13/38,871 (0.03%) (Torene et al., 2020) by MEIs. As these findings are limited to coding regions of the genome, the diagnostic rate should increase as MEIs in non‐coding regions become more interpretable. Even though the diagnostic rate seems low, if the variant calling algorithm is optimized, performing an additional analysis to discover MEIs from the existing exome data may be relatively easier than performing alternate genetic tests and if it can give a conclusive answer to even few patients, it could be considered worthwhile for laboratories to implement MEI caller as part of the routine informatics pipeline.

## 5 CONCLUSION

In conclusion, this case report illustrates a LINE‐1 insertion identified in LDLR causing a disease by disrupting coding regions of the gene. The finding highlights the importance of using multiple variant callers such as MEI callers to increase the diagnostic rate. Also, it adds a new variant type to the *LDLR* disease‐causing variant catalog which can be useful to patients with unknown genetic etiology or subject to screening.

## AUTHOR CONTRIBUTIONS

Yongjun Song carried out WES analysis, wrote a draft, and revised the report. Reham Abdel Haleem Abo Elwafa and Omneya Magdy Omar examined the family who participated and collected samples. Go Hun Seo provided medical supervision. Hane Lee assisted in reviewing this paper and managed the project.

## CONFLICT OF INTEREST STATEMENT

The authors declare no conflict of interest.

## ETHICS STATEMENT AND PATIENT CONSENT STATEMENT

This study was approved by the Alexandria Faculty of Medicine Ethics Committee (IRB code 00012098‐FWA: No. 00018699. Ethics approval number: No. 0305926) and the patient and her family were consented for the study.

## Data Availability

The data that support the findings of this study are available on request from the corresponding author. The data are not publicly available due to privacy or ethical restrictions.
